# What Factors Would Make Single-Vehicle Motorcycle Crashes Fatal? Empirical Evidence from Pakistan

**DOI:** 10.3390/ijerph19105813

**Published:** 2022-05-10

**Authors:** Amjad Pervez, Jaeyoung Lee, Helai Huang, Xiaoqi Zhai

**Affiliations:** 1 School of Traffic and Transportation Engineering, Central South University, Changsha 410075, China; amjadpervez04@csu.edu.cn (A.P.); huanghelai@csu.edu.cn (H.H.); 2 Department of Civil, Environmental and Construction Engineering, University of Central Florida, Orlando, FL 32816, USA; 3 School of Civil Engineering, Zhengzhou University, Zhengzhou 450001, China; xiaoqizhai@zzu.edu.cn

**Keywords:** motorcycle safety, injury severity, single-vehicle crashes, unobserved heterogeneity, random parameter model, heterogeneity in means and variances

## Abstract

The existing research on motorcycle safety has shown that single-vehicle motorcycle crashes (SVMC) account for a higher fatality rate than other types of crashes. Also, motorcycle safety has become one of the critical traffic safety issues in many developing countries, such as Pakistan, due to the growing number of motorcycles and lack of sufficient relevant infrastructure. However, the available literature on SVMC and motorcycle safety in developing countries is limited. Therefore, the present study attempted to investigate the factors that contribute to the injury severity of SVMC in a developing country, Pakistan. For this purpose, a random parameter logit model with heterogeneity in means and variances is developed using two years of data extracted from the road traffic injury research project in Karachi city, Pakistan. The study’s findings show that the presence of pillion passengers and young motorcyclists indicators result in random parameters with heterogeneity in their means and variances. The study’s results also reveal that the summer, morning time, weekends, older motorcyclists, collisions with fixed objects, speeding, and overtaking are positively, while younger motorcyclists and the presence of pillion passengers are negatively associated with fatal crashes. More importantly, in the particular Pakistan’s context, female pillion passenger clothes trapped in the wheel, riding under the influence, intersections, U-turns, and collisions due to loss of control are also found to significantly influence the injury severity of SVMC. Based on these research findings, multiple appropriate countermeasures are recommended to enhance motorcycle safety in Pakistan and other developing countries with similar problems.

## 1. Introduction

Numerous research studies on road safety have been carried out in developed countries, using large datasets and rigorous methodologies to better understand the crash mechanism and provide appropriate countermeasures in terms of road design, education, and outreach. In contrast, developing countries lack resources that limit their ability to do such research. Thus, mitigation initiatives typically rely on qualitative techniques, such as expert views, which may be affordable but ineffective since biased results might exacerbate safety concerns. However, as the world moves toward big data availability, new datasets are becoming available in developing countries, and modern approaches may greatly assist safety research and enhance our understanding of traffic safety worldwide.

Globally, in road traffic crashes, around 1.35 million people die every year. Developing countries account for approximately 93% of these road fatalities while having just 60% of the total registered vehicles on the roads [1]. The burden of traffic crashes in developing economies is three times higher than that of developed economies. Pakistan is a lower-middle-income country, with approximately 25,781 traffic fatalities annually [1]. Likewise, the estimated death rate due to traffic crashes per 100,000 population in the country is 14.3 [1]. Moreover, traffic crashes and injuries result in economic losses of more than 100 billion rupees (equivalent to ~1.3 billion US dollars) [2]. Therefore, there is an immense need to explore different traffic safety aspects in Pakistan to improve safety, which will, in turn, reduce the economic burden.

Like other developing countries, in Pakistan, the common mode of transportation includes cars, busses, mini-busses, auto-rickshaws, and motorcycles. However, in the last decade, the ownership and use of motorcycles have substantially increased in the country. For instance, the registered number of motorcycles has surged from 4.3 million to 21.9 million between 2010 and 2019, accounting for 75.5% of all motorized vehicles in 2019 [3]. Such a rapid increase in motorcycles is associated with road and safety issues, including traffic congestion, parking problems, and road crashes. Moreover, in Pakistan, due to the lack of specific motorcycle lanes, motorcyclists share the same infrastructure with other motor vehicle users, making them more prone to be seriously injured compared to other countries [4,5]. For instance, motorcyclists are involved in more than 45% of all traffic crashes in Pakistan’s largest city, Karachi [6].

Despite the serious safety concerns raised by the motorcycles, there exists limited literature on motorcycle safety in Pakistan. Most of the existing studies in the country have concentrated on the investigation of motor vehicle crashes [7,8,9] and road safety issues [10,11,12], while only a few recent studies [4,5] have attempted to investigate the motorcyclists’ injury severities. However, to the authors’ best knowledge, after a thorough literature review, no study on single-vehicle motorcycle crashes (SVMC) in Pakistan has been published to date. Although studies [13,14,15,16,17,18,19] have been conducted in developed countries to investigate SVMC, their findings may not be directly applicable in the Pakistan context due to the differences in the drivers’ behavior, roadway environment, and safety policies. Also, in Pakistan, due to the lack of sufficient relevant infrastructure and substantial increase in the number of motorcycles, there are significant chances that the issues surrounding motorcyclists might increase in the future. Moreover, further research focusing on the severity of motorcycle crashes in various geographical locations is required to average the regional bias associated with motorcycle crash severity.

The objective of this research is to gain a clearer understanding of the potential risk factors that contribute to SVMC. Based on a comprehensive dataset from Karachi city, containing 16,179 SVMC, a random parameter model with heterogeneity in means and variances is developed to identify the factors influencing the injury severity of SVMC. In addition, based on the current study’s findings, numerous safety-specific countermeasures for enhancing motorcycle safety in Pakistan and other developing countries with similar problems are recommended.

## 2. Literature Review

The existing literature [1,20,21] suggests that motorcycle crashes are generally more severe than car crashes. This is primarily because motorcyclists lack protection in a crash event [21,22]; also, they are more likely to take risks, resulting in an increase in the number of motorcycle-related fatalities [20].

Among the motorcycle crashes, studies [16,23,24] have concluded that single-vehicle crashes have a higher fatality rate compared to other types of crashes. However, only a few studies [13,14,15,16,17,18,19] have attempted to examine the pattern, mechanism, and risk factors of SVMC. These studies have concluded that a number of variables could affect the severity of SVMC. The significant variables can be classified into five categories: motorcyclist (a person who operates the motorcycle), vehicle, crash, roadway, and environmental characteristics. Motorcyclist characteristics, including age, gender, riding under the influence of alcohol, and helmet use, were found to influence the severity of SVMC [13,14,15,16,17]. Vehicle characteristics, such as motorcycle size, type, and production year, were likely to contribute to the severity of SVMC [15]. Also, it has been concluded that the crash characteristics, such as exceeding speed limits, rollover crashes, and collisions with fixed objects and animals, could impact the severity of SVMC [13,15,16,19]. Finally, the contributory factors related to roadway and environmental characteristics include curvature, roadway segment, road surface type, lighting, visibility conditions, day of the week, and season [13,14,15,16,19].

Methodologically, the motorcyclists’ injury severities have been investigated through various modeling approaches, including the binary logit model [25,26,27], ordered probit/logit model [24,28,29], and multinomial logit model [30,31]. However, the conventional models overlook the unobserved heterogeneity arising from the unavailability of certain key attributes in the crash data, leading to biased parameter estimation and erroneous conclusions [32]. Therefore, random parameter models are introduced to solve the issue of unobserved heterogeneity in the motorcyclists’ injury severity analysis [4,19,33,34]. These models can capture the heterogeneous effect of the explanatory variables by allowing them to vary across individual outcomes. To allow further flexibility to the random parameter models to account for the unobserved heterogeneity resulting from the factors affecting the random parameters’ means and variances, studies have developed random parameter models with heterogeneity in means and variances to assess motorcyclists’ injury severity [5,17,18].

Since limited research efforts have been conducted to understand the mechanism and risk factors related to SVMC, particularly in developing countries. Therefore, investigating the factors affecting SVMC in a developing country (Pakistan) appears to be beneficial by providing useful evidence for resolving motorcyclists’ safety issues. Moreover, to capture the complex layers of unobserved heterogeneity, a random parameter model with heterogeneity in means and variances can be employed to provide clear insight into the factors influencing the injury severity of SVMC, which will help in devising effective countermeasures to enhance motorcycle safety in a developing country such as Pakistan.

## 3. Data Description

**Figure 1:**
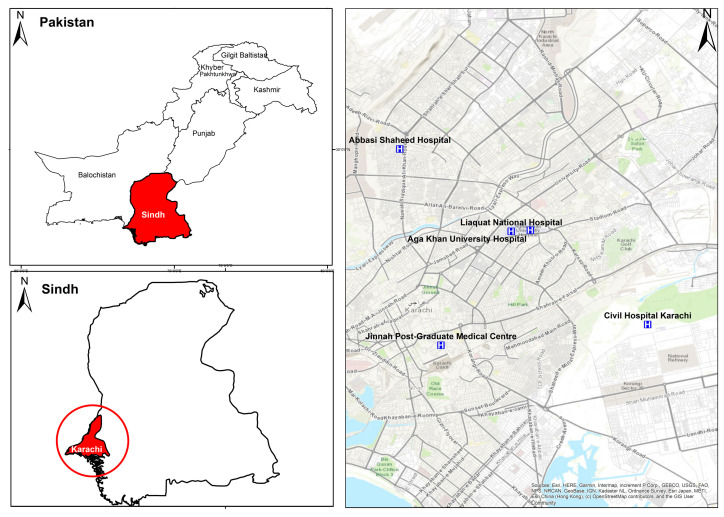
Location of the study area.

The study area for this research is Karachi, the most populous and largest city in Pakistan [3]. Motorcycle is one of the primary transportation modes in Karachi city, constituting over two-thirds of all the registered vehicles [6]. The crash information was extracted from the road traffic injury research project. The project was set up in the emergency of five hospitals (refer to Figure 1), namely, Abbasi Shaheed Hospital, Civil Hospital Karachi, Aga Khan University Hospital, Jinnah Post-Graduate Medical Centre, and Liaquat National Hospital. These hospitals were selected as they cover and attend almost half to three-quarters of all the traffic crashes in Karachi [6,35].

In this study, only the SVMC in Karachi city between 1 January 2014 and 31 December 2015 were considered for analysis. Overall, 16,179 crashes were recorded in the final dataset. Only 354 of the selected crashes had fatal outcomes (i.e., immediate or subsequent death from injuries within seven days after a crash), while the remainder resulted in non-fatal injuries. As a result, the injury severity was divided into fatal and non-fatal injury outcomes. The dataset also contains detailed information on the environmental, motorcyclist, motorcycle, and roadway characteristics. The variables for the model analysis were chosen using two criteria: including characteristics that have been studied previously in the literature and using variables based on the local environment to establish other factors that could potentially impact the motorcyclists’ injury severity [22]. Finally, 14 explanatory variables were retained and divided into four groups, namely, temporal, motorcyclist, roadway, and crash characteristics. The explanatory factors that were found statistically significant in the models assessing the motorcyclists’ injury severity in SVMC are summarized in Table 1.

## 4. Methodology

### 4.1. Model Estimation

Researchers have attempted to employ various models with accurate and unbiased inferences for motorcycle injury severity, including random parameter multinomial logit model [36,37,38], latent-class random parameter model [39,40], random parameter model with heterogeneity in means [41], and random parameter model with heterogeneity in means and variances [5,18]. In the current study, the injury severities of the SVMC were investigated using two distinct injury severity outcomes (i.e., fatal versus non-fatal); therefore, a random parameter binary logit model, with heterogeneity in means and variances, was developed to assess the factors affecting the injury severities of SVMC. Also, a random parameter model was developed for model comparison.

The standard binary logit model can be represented as follows [42]:

$$Y_{i}\sim Binomial\left(P_{i},N\right)$$

$$logit\left(P_{i}\right)=\log\left(\frac{P_{i}}{1-P_{i}}\right)=\beta_{ο}+\overset{K}{\underset{k=1}{\sum }}\beta_{k}X_{ik}$$

where $P_{i}$ denotes the probability of $Y_{i}=1$ (fatal outcome) and $1-P_{i}$ is the probability of $Y_{i}=0$ (non-fatal outcome). $X_{ik}$ is the kth independent variable (k=1, 2,…, K). $\beta_{ο}$ is the model constant and $\beta_{k}$ are the parameter estimates corresponding to $X_{ik}$.

The abovementioned basic model has the disadvantage of assuming the parameters to be invariable, implying that the influence of each factor on injury severity is constant across observations. Consequently, the model ignores the influence of unobserved heterogeneity. Thus, in order to examine the impact of unobserved heterogeneity, the parameters are allowed to vary across individual outcomes. In addition, unobserved heterogeneities in the random parameters’ means and variances are accounted for by allowing the parameters to be a vector of the estimable parameters. Accordingly, the parameters’ coefficients can be formulated as follows [5,43]:

$$\beta_{i}=\beta +\theta_{i}Z_{i}+\sigma_{i}EXP{\left(\omega_{i}W_{i}\right)}_{v_{i}}$$

where β indicates the mean estimated parameter across crashes, and $Z_{i}$ denotes the explanatory variables’ vector capturing heterogeneities in the mean with parameter vector $\theta_{i}$. $W_{i}$ represents the explanatory variables’ vector capturing heterogeneities in the standard deviation $\sigma_{i}$ with parameter vector $\omega_{i}$ and $v_{i}$ is an error term.

The statistical package NLOGIT5 was utilized to estimate the model via 1000 Halton draws [44]. Several random parameter distributions, including uniform, triangular, log-normal, and normal, were explored in this study. Finally, the normal distribution was chosen to fit the final model as it produces statistically superior results [45,46].

### 4.2. Model Comparison

For the comparison and model adjustment between different models, the Akaike information criterion (AIC) and McFadden’s $\rho^{2}$ were utilized. The AIC represents a measure of the balance between the model bias and variance by comparing the parameters’ number (K) and model likelihood (L), as follows:

AIC=−2ln(L)+2K

McFadden’s $\rho^{2}$ compares the likelihood estimated for the intercept-only model {LL(0)} to that of the proposed model {LL(β)}, as follows:

$$\rho^{2}=1-\frac{LL\left(\beta \right)}{LL\left(0\right)}$$

### 4.3. Marginal Effects

The marginal effects were calculated to better quantify the effect of the significant explanatory variables (X) on the outcome probability of injury severity $\left(P_{i}\right)$. The marginal effects represent the influence of a one-unit change in any explanatory variable ($X_{ik}$) on the probability to result in the injury severity $\left(P_{i}\right)$. More specifically, a marginal effect for an indicator variable ($X_{ik}$) is the probability difference when the indicator variable changes from zero to one [42,47].

$$ME_{X_{ik}}^{P_{i}}=P_{i}\left[X_{ik}=1\right]-P_{i}\left[X_{ik}=0\right]$$

## 5. Results

First, to prevent multicollinearity problems, variance inflation factors were computed. For the age groups <25 and 25–40, the variance inflation factor has a maximum value of 2.17, suggesting no substantial multicollinearity among the explanatory variables. Second, to ensure that every added variable substantially improves the model’s efficiency, the likelihood ratio test was used. In addition, to concentrate on the statistically significant variables, only those with a significance level of 10% or below were retained in the final model. Third, if the parameters resulted in statistically significant standard deviations, they were regarded as random parameters. On the other hand, the parameters remained fixed. Finally, after estimating the random parameter binary logit model, heterogeneities in the random parameters’ means and variances were further explored. All the significant variables were tested to explore whether they had any significant effect on the random parameters’ means and variances.

**Figure 2:**
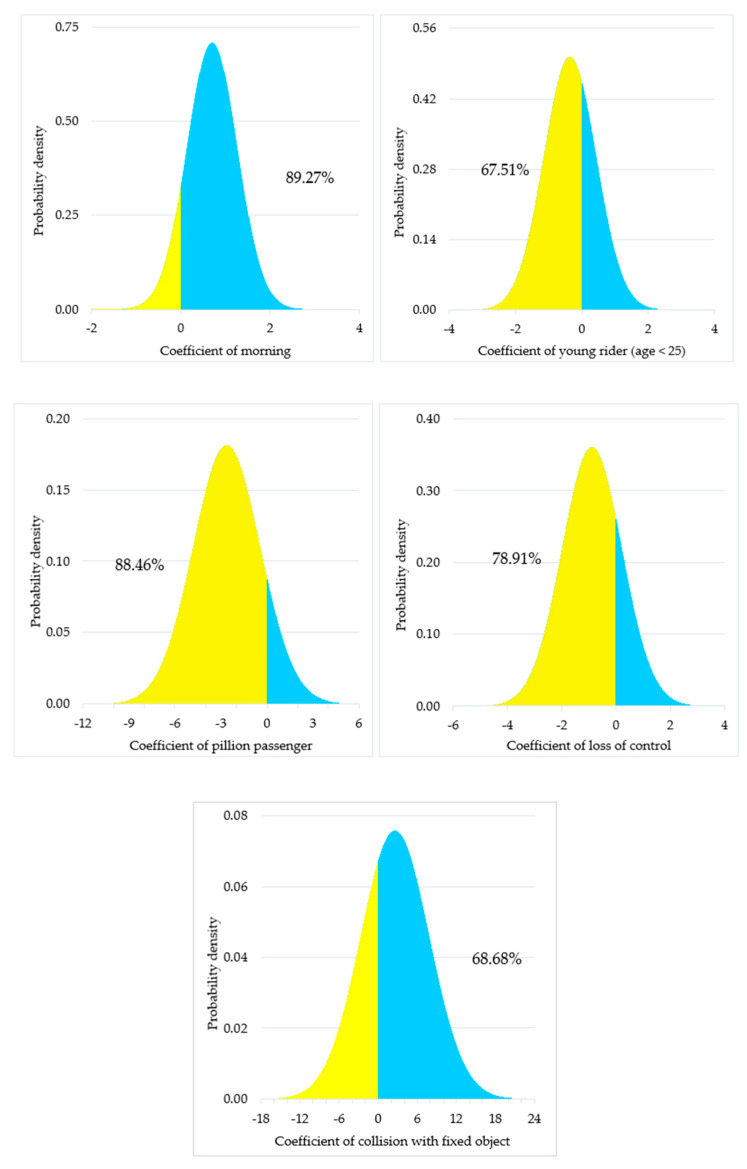
Distribution of the random parameters.

Table 2 summarizes the estimated models’ overall goodness-of-fit. As shown in the table, the random parameter model with heterogeneity in means and variances has a higher McFadden’s $\rho^{2}$ value than the random parameter model; thus, it fits the data well [48]. Also, it has a lower AIC value (2794.12) than the random parameter model (2800.57). Moreover, the random parameter model with heterogeneity in means and variances is statistically better (*p* = 0.006) at the 95% confidence level based on the likelihood ratio test. Therefore, the results and discussions presented in Table 3 and subsequent sections are based on this model. The marginal effects for each explanatory variable are also provided in Table 3. In this study, the effect of factors, including morning, younger motorcyclists, pillion passengers, collisions due to loss of control, and fixed objects, can be best quantified when considered as random variables, and the corresponding normal distributions are illustrated in Figure 2.

## 6. Discussions

### 6.1. Heterogeneity in Means and Variances

Considering the heterogeneities in the means and variances, the present study found that the indicator of the pillion passengers results in random parameters with heterogeneity in means and variances, while the indicator of young motorcyclists (age < 25) produces random parameters with heterogeneity in means only. As shown in Table 3, the means of the pillion passengers indicator increases if the clothes of the female pillion passengers are trapped in the wheel of the motorcycle, suggesting an increase in fatal injuries. In the context of Pakistan, this finding is intuitive as females usually wear long clothes, and when the pillion passenger is female, their long clothes can easily trap in the chain or rear wheel of the motorcycle, thus increasing the risk of fatal injury crashes. Another variable, “speeding”, also increased the mean of the indicator parameter “pillion passengers”. It is because speeding results in higher fatal crashes involving a motorcycle [49], and when there is a pillion passenger, the probability of fatalities increases due to a greater number of people. Similarly, speeding also increases the mean of the indicator of the young motorcyclists (age < 25). It could be attributed to the fact that young motorcyclists lack experience and also like to take risky behaviors, including speeding, which increases the risk of fatal injury crashes involving young motorcyclists exceeding speed limits. Concerning the explanatory variables that can influence the random parameters’ variances, speeding tended to influence the variance of the indicator of the pillion passengers only.

### 6.2. Temporal Characteristics

In terms of the season variable, SVMC that occurred during the summer season has a 6% higher likelihood of resulting in fatal crashes. This could be because, in Karachi city, the summer season is associated with relatively high temperature and humid climate, leading to substantial visual impairment. In addition, due to the thermal discomfort, motorcyclists are less likely to use helmets, increasing the probability of fatal injury outcomes [15,46,50]. Moreover, in summer, the monsoon season reaches its peak in the study area [51], resulting in poor road surface conditions (i.e., wet road surfaces and drainage problems) and visibility, all of which significantly increase the likelihood of fatal crashes [52,53,54].

Concerning the day of the week, SVMC that occurred on weekends were found to cause more fatal crashes. This might be because on weekends, owing to the holidays, the motorcyclists like to hang around on the roads, and the traffic volume is relatively lower. As a result, the motorcyclists engage in various risk-taking behaviors, including drifting, reckless driving, and speeding, increasing the chance of fatal injuries. The result is in line with prior studies from other lower- and middle-income countries [31,55,56].

In terms of the time of day, morning time (06:00–08:59) is associated with an increased likelihood of fatal injuries in SVMC, with a probability of about 3%. It might be because, during the early morning hours, motorcyclists prefer to ride faster in order to reach their workplace or school on time, and higher speeds are related to fatal injury outcomes. The finding is supported by previous literature [57,58]. The coefficient corresponding to the morning variable follows a normal distribution (mean = 0.69, standard deviation = 0.56), implying that the early morning hours increase the chances of fatal crashes by 89.27%.

### 6.3. Motorcyclist Characteristics

The study found that SVMC that involve younger motorcyclists (aged < 25) are less likely to be fatally injured. The likelihood of fatal injuries decreases by 2% whenever a young motorcyclist is involved in a single-vehicle motorcycle crash. This might stem from young motorcyclists’ faster reactions because of their physical flexibility [59,60]. The result is also consistent with existing literature [13,14,15,16] on SVMC. For the variable “young age”, the normal distribution is supported by its mean value of −0.36 and standard deviation of 0.79. These results indicate that in 67.51% of the crash observations, young motorcyclists are less likely to sustain fatal injuries (see Figure 2). The findings of the study also revealed that when older motorcyclists (age ≥ 55) are involved in a single-vehicle motorcycle crash, they have a 4% higher chance of being fatally injured. This finding is in line with the existing research [15,30], as older motorcyclists take longer to respond to hazardous situations, and their physiological condition is deteriorating.

When it comes to the presence of pillion passengers, the study discovered that SVMC involving pillion passengers result in 16% lower fatal injury outcomes. One probable explanation is that in the presence of pillion passengers, motorcyclists are more cautious since the mass of the motorcycle increases. Also, in Pakistan, motorcycle is one of the primary transportation modes, and the pillion passenger is usually a family member or friend; thus, motorcyclists tend to drive more cautiously when they are present. Several prior investigations [15,61,62] have also found that crashes involving a pillion passenger reduce the risk of fatal injuries. The variable “pillion passengers” also yields a coefficient with a normal distribution having a mean of −2.63 and a standard deviation of 2.19, suggesting that 88.46% of the pillion passengers will have a lower likelihood of fatal injury outcomes.

The study’s results indicated that SVMC that involve female pillion passengers whose clothes are trapped in the wheel have a higher risk of fatalities (by 3%). This is an interesting finding as the variable “female pillion passengers’ clothes trapped in the wheel” is unique in Pakistan’s context. The increased likelihood of this kind of crash is due to the fact that females in Pakistan typically wear loose garments such as long, flowing overcoats (abayas/burqas) or long shirts (kameez) over trousers (shalwar). Also, they sit on the motorcycle in a side-way manner. Therefore, their clothes can easily get trapped in the chain or rear wheel of the motorcycle [63], leaving both the motorcyclist and passenger more vulnerable to fatal injuries [64].

Crashes involving motorcyclists under the influence of drugs or alcohol result in 2% higher fatal injuries. Drugs or alcohol impair the motorcyclist’s ability to assess road conditions and prevent them from fully controlling the motorcycle, increasing the chances of risky maneuvers [65]. The result is consistent with earlier research [13,14,16] reporting that riding under the influence increases the risk of fatal injuries in SVMC.

### 6.4. Roadway Characteristics

Regarding the roadway segment, the intersection significantly increases the probability of fatal injuries by 5% in SVMC. The finding is not in accord with the existing research [14,15,16] on SVMC, which interpreted that crashes at intersections are less likely to cause fatal injuries since motorcyclists generally reduce their speeds near the intersection. This might be because, in Pakistan, due to the unavailability of adequate signals and cameras at intersections [66], motorcyclists are usually involved in various unsafe behaviors such as aggressive driving, abrupt lane changes, and sudden braking, which, in turn, result in a higher probability of fatal crashes [67,68].

### 6.5. Crash Characteristics

Regarding the crash causes, SVMC related to speeding and overtaking result in increased fatal injury outcomes with probabilities of approximately 5% and 1%, respectively. In the case of speeding, the finding is not surprising since when the motorcyclists are involved in speeding, they have less control over the motorcycle and minimal time to react in a hazardous situation. Similarly, for overtaking, the higher fatality rate could be attributed to the fact that since roads in Pakistan do not have separate motorcycle lanes, motorcyclists use the same lane as heavy vehicles [5]. Thus, during overtaking, even a slight motorcycle-to-vehicle contact may result in serious injuries to the motorcyclists. The finding is consistent with previous research on SVMC [13,14,15]. Finally, the U-turn indicator also increases the risk of fatal injury outcomes by 3% in SVMC. It could be attributed to the fact that, in Pakistan, motorcyclists make U-turns at unspecified locations. Consequently, they are more exposed to various dangerous interactions and are likely to be involved in more side-impact collisions with approaching vehicles, leading to fatal injury motorcycle crashes [69].

In terms of the collision types, collisions with fixed objects result in a 3% higher probability of fatal injuries. As expected, during a collision, fixed objects are unlikely to absorb much energy, thus resulting in higher fatalities among motorcyclists. Prior investigations [14,15] have also concluded that collisions with fixed objects cause more serious injuries to the motorcyclists. The variable “collisions with fixed objects” has a normally distributed coefficient (mean = 2.56 and standard deviation = 5.26). Given the distributional values, it can be interpreted that 68.68% of the fixed object collisions tend to increase fatal injuries to the motorcyclists. The study also discovered that collisions due to loss of control decrease the risk of fatal injury outcomes by 2%. Similar finding has been concluded by a previous study [70] on SVMC. The loss of control variable yielded a normally distributed parameter (mean = −0.88, standard deviation = 1.11), indicating that loss of control crashes has a 78.90% lower risk of fatal collisions.

## 7. Conclusions

Motorcycle safety has become one of the pressing traffic safety issues in many developing countries such as Pakistan due to the growing number of motorcycles in recent years. Therefore, this research was conducted to investigate the influence of various factors on the fatality of SVMC in Pakistan. A random parameter model with heterogeneity in means and variances was estimated to evaluate the SVMC in Karachi city, Pakistan. The factors included the motorcyclists’ socio-demographics, environmental characteristics, crash characteristics, and roadway features.

According to the findings of this study, taking into account the heterogeneity in the random parameters’ means and variances enhances the overall model fitness and provides new insights into the injury severity of SVMC. It was found that the presence of pillion passengers and young motorcyclist indicators have a varying effect on the SVMC. The heterogeneity in the random parameters’ means and variances regarding the pillion passengers and young motorcyclists indicators were found to be related to female pillion passengers whose clothes were stuck in the wheel and speeding.

Apart from the above, the study’s findings revealed that the summer season has a higher risk of fatal crashes. The likelihood of fatal crash occurrence is relatively higher during the early morning hours. The probability of fatal injury increases when crashes occur on weekends. Crashes involving young motorcyclists tend to have a lower, while those involving older motorcyclists have a higher risk of fatal injuries. The presence of pillion passengers causes a lower chance of fatal crashes. SVMC that involve a female pillion passenger whose clothes got trapped in the motorcycle or motorcyclist under the influence result in fatal injuries. The research results reveal that crashes that occurred at intersections result in higher fatal injury outcomes. It has been found that motorcyclist violations, including speeding, overtaking, and U-turns, increase the fatal injury outcomes in SVMC. Finally, collisions with fixed objects result in higher while loss of control in lower fatal crashes.

According to the current research findings, countermeasures to decrease SVMC in Pakistan are suggested. The conventional 4Es approach, i.e., enforcement, engineering, education, and emergency response, is adopted to develop the safety-specific measures. Enforcement measures, including strict speed enforcement (particularly at the intersections) and riding under the influence policies, to reduce the speeding and riding under the influence crashes, respectively, are suggested. Engineering measures, such as periodic maintenance of roads and drainage systems to minimize the crashes during the summer season (monsoon season), and provision of exclusive motorcycle lanes to lower the overtaking crashes are recommended. Similarly, providing wider shoulders to reduce motorcycle–fixed-object crashes and designing appropriate coverings for the motorcycle’s back wheel and chains to prevent female clothes from being trapped in the motorcycle are advised. Education measures, including arranging formal motorcyclist training programs for the motorcyclists while specifically focusing on the young motorcyclists, awareness regarding injuries caused by loose clothes, and public information campaigns about motorcycle safety, are suggested. Regarding the emergency measures, provision and improvement in the emergency medical services (e.g., rescue 1122) and emergency management systems can help increase the survivability of the injured motorcyclists.

The current study’s contribution is to provide insight into the risk patterns of SVMC in a developing country. In addition, the present study’s findings are expected to raise concerns and discussions about motorcycle safety in Pakistan. Meanwhile, the authorities could utilize the research findings and suggested countermeasures to enhance motorcycle safety in the country. However, it should also be noted that the available data constrain the findings of the present study. For instance, the road traffic injury surveillance system does not contain important variables such as underreporting of crashes and weather information. Thus, more comprehensive data should be used to confirm and extend the findings of the present study in the future.

## Figures and Tables

**Table 1:** Descriptive statistics of the significant explanatory variables.

Variable Description	Frequency	Percentage
Non-fatal	15,825	97.81
Fatal	354	2.19
**Temporal Characteristics**		
Summer season indicator (If a crash happens in summer = 1, otherwise = 0)	4759	29.41
Weekend indicator (If a crash happens on the weekend = 1, otherwise = 0)	5052	31.23
Morning indicator (If a crash happens between 06:00–08:59 = 1, otherwise = 0)	818	5.06
**Motorcyclist Characteristics**		
Younger motorcyclist indicator (If the age of a motorcyclist is less than 25 years = 1, otherwise = 0)	8460	52.29
Older motorcyclist indicator (If the age of a motorcyclist is equal/more than 55 years = 1, otherwise = 0)	1072	6.62
Pillion passenger indicator (If a pillion passenger is present = 1, otherwise = 0)	2297	14.20
Female pillion passenger’s clothes indicator (If the clothes of a female pillion passenger got stuck in the wheel/chain = 1, otherwise = 0)	802	4.96
Riding under the influence indicator (If a motorcyclist is riding under the influence of drugs or alcohol =1, otherwise = 0)	1232	7.61
**Roadway Characteristics**		
Intersection indicator (If a crash happens at intersection = 1, otherwise = 0)	2836	17.53
**Crash Characteristics**		
Speeding indicator (If a motorcyclist is exceeding speed limit = 1, otherwise = 0)	1957	12.10
Wrong overtaking indicator (If a motorcyclist is taking wrong overtake = 1, otherwise = 0)	1363	8.42
U-turn indicator (If a motorcyclist is making a U-turn = 1, otherwise = 0)	1470	9.09
Fixed objects indicator (If a crash is fixed object type = 1, otherwise = 0)	2289	14.15
Loss of control indicator (If a crash loss of control type = 1, otherwise = 0)	2118	13.09

**Table 2:** Comparison of the estimated models’ goodness-of-fit.

Model	*RPL*	*RPL_HMV*
Observations	16,179	16,179
Parameters	20	24
LL(0)	−1548.74	−1548.74
LL(β)	−1380.28	−1373.06
AIC	2800.57	2794.12
$\mathrm{McFadden}’s\;\mathrm{pseudo}\;\left(\rho^{2}\right)$	0.11	0.12
$\chi^{2}=$−2 [LL(RPL)−LL(RPL_HMV)]	14.46	
Degree of freedom	4	
p-value	0.006	

**Table 3:** Estimated model results.

Variable	Coefficient	*p*-Value	Marginal Effects
Constant	−3.06	<0.001	-
**Temporal Characteristics**			
Summer season indicator (If a crash happens in summer = 1, otherwise = 0)	0.21	0.023	0.06
Weekend indicator (If a crash happens on the weekend = 1, otherwise = 0)	0.18	0.040	0.04
Morning indicator (If a crash happens between 06:00–08:59 am = 1, otherwise = 0)	0.69	<0.001	0.03
*Std. dev. Morning indicator*	0.56	0.004	-
**Motorcyclist Characteristics**			
Younger motorcyclist indicator (If age of the motorcyclist is less than 25 years = 1, otherwise = 0)	−0.36	<0.001	−0.19
*Std. dev. Young motorcyclist indicator*	0.79	0.0350	-
Older motorcyclist indicator (If age of the motorcyclist is equal/more than 55 years = 1, otherwise = 0)	1.16	<0.001	0.04
Pillion passenger indicator (If a pillion passenger is present = 1, otherwise = 0)	−2.63	<0.001	−0.16
*Std. dev. Pillion passenger indicator*	2.19	<0.001	-
Female pillion passenger’s clothes indicator (If the clothes of a female pillion passenger got stuck in the wheel/chain = 1, otherwise = 0)	0.51	0.017	0.03
Riding under the influence indicator (If a motorcyclist is riding under the influence of drugs or alcohol = 1, otherwise = 0)	0.56	0.022	0.02
**Roadway Characteristics**			
Intersection indicator (If a crash happens at intersection = 1, otherwise = 0)	0.28	0.005	0.05
**Crash Characteristics**			
Speeding indicator (If a motorcyclist is exceeding speed limit = 1, otherwise = 0)	0.41	<0.001	0.05
Wrong overtaking indicator (If a motorcyclist is taking wrong overtake=1, otherwise = 0)	0.45	0.029	0.01
U-turn indicator (If a motorcyclist is making a U-turn = 1, otherwise = 0)	0.63	<0.001	0.03
Fixed objects indicator (If a crash is fixed object type = 1, otherwise = 0)	2.56	0.012	0.03
*Std. dev. Fixed objects indicator*	5.26	<0.001	-
Loss of control indicator (If a crash loss of control type = 1, otherwise = 0)	−0.88	<0.001	−0.02
*Std. dev. Loss of control indicator*	1.11	0.031	-
**Heterogeneity in the random parameters’ means**			
Pillion passengers: Female pillion passenger’s clothes indicator (If clothes of a female pillion passenger got stuck in the wheel/chain = 1, otherwise = 0)	1.35	0.005	-
Pillion passengers: Speeding indicator (If a motorcyclist is exceeding speed limit = 1, otherwise = 0)	1.26	0.003	-
Young motorcyclist: Speeding indicator (If a motorcyclist is exceeding speed limit = 1, otherwise = 0)	1.93	0.015	-
**Heterogeneity in the random parameters’ variances**			
Pillion passengers: Speeding indicator (If a motorcyclist is exceeding speed limit = 1, otherwise = 0)	1.48	0.007	-

## Data Availability

Not applicable.

## References

[1] WHO (2018). Global Status Report on Road Safety 2018: Supporting a Decade of Action.

[2] Ahmed A. (2007). Road Safety in Pakistan.

[3] Pakistan Bureau of Statistics (2019). Pakistan Statistical Yearbook Pakistan Bureau of Statistics.

[4] Pervez A., Lee J., Huang H. (2021). Identifying Factors Contributing to the Motorcycle Crash Severity in Pakistan. J. Adv. Transp..

[5] Waseem M., Ahmed A., Saeed T.U. (2019). Factors affecting motorcyclists’ injury severities: An empirical assessment using random parameters logit model with heterogeneity in means and variances. Accid. Anal. Prev..

[6] Shamim M.S., Razzak J.A., Jooma R., Khan U.R. (2011). Initial results of Pakistan’s first road traffic injury surveillance project. Int. J. Inj. Control Saf. Promot..

[7] Hyder A.A., Ghaffar A., Masood T.I. (2000). Motor vehicle crashes in Pakistan: The emerging epidemic. Inj. Prev..

[8] Azam K., Shakoor A., Shah R.A., Khan A., Shah S.A., Khalil M.S. (2014). Comparison of fatigue related road traffic crashes on the national highways and motorways in Pakistan. J. Eng. Appl. Sci..

[9] Hammad H.M., Ashraf M., Abbas F., Bakhat H.F., Qaisrani S.A., Mubeen M., Fahad S., Awais M. (2019). Environmental factors affecting the frequency of road traffic accidents: A case study of sub-urban area of Pakistan. Environ. Sci. Pollut. Res..

[10] Ullah H., Farooq A., Shah A.A. (2021). An Empirical Assessment of Factors Influencing Injury Severities of Motor Vehicle Crashes on National Highways of Pakistan. J. Adv. Transp..

[11] Rashid H.M., Ahmed A., Wali B., Qureshi N.A. (2018). An analysis of highway work zone safety practices in Pakistan. Int. J. Inj. Control Saf. Promot..

[12] Batool Z., Carsten O. (2018). Attitudinal segmentaion of drivers in Pakistan: The potential for effective road safety campaigns. Accid. Anal. Prev..

[13] Shankar V., Mannering F. (1996). An exploratory multinomial logit analysis of single-vehicle motorcycle accident severity. J. Saf. Res..

[14] Shaheed M.S., Gkritza K. (2014). A latent class analysis of single-vehicle motorcycle crash severity outcomes. Anal. Methods Accid. Res..

[15] Savolainen P., Mannering F. (2007). Probabilistic models of motorcyclists’ injury severities in single- and multi-vehicle crashes. Accid. Anal. Prev..

[16] Farid A., Ksaibati K. (2021). Modeling severities of motorcycle crashes using random parameters. J. Traffic Transp. Eng. (Engl. Ed.).

[17] Islam M. (2021). The effect of motorcyclists’ age on injury severities in single-motorcycle crashes with unobserved heterogeneity. J. Saf. Res..

[18] Alnawmasi N., Mannering F. (2019). A statistical assessment of temporal instability in the factors determining motorcyclist injury severities. Anal. Methods Accid. Res..

[19] Xin C., Wang Z., Lee C., Lin P.-S. (2017). Modeling Safety Effects of Horizontal Curve Design on Injury Severity of Single-Motorcycle Crashes with Mixed-Effects Logistic Model. Transp. Res. Rec. J. Transp. Res. Board.

[20] Chen C.-F. (2009). Personality, safety attitudes and risky driving behaviors—Evidence from young Taiwanese motorcyclists. Accid. Anal. Prev..

[21] Lin M.-R., Kraus J.F. (2009). A review of risk factors and patterns of motorcycle injuries. Accid. Anal. Prev..

[22] Rifaat S.M., Tay R., de Barros A. (2012). Severity of motorcycle crashes in Calgary. Accid. Anal. Prev..

[23] Zhou M., Chin H.C. (2019). Factors affecting the injury severity of out-of-control single-vehicle crashes in Singapore. Accid. Anal. Prev..

[24] Pai C.-W., Saleh W. (2007). An analysis of motorcyclist injury severity under various traffic control measures at three-legged junctions in the UK. Saf. Sci..

[25] Haque M.M., Chin H.C., Huang H. (2009). Modeling fault among motorcyclists involved in crashes. Accid. Anal. Prev..

[26] Pai C.-W. (2009). Motorcyclist injury severity in angle crashes at T-junctions: Identifying significant factors and analysing what made motorists fail to yield to motorcycles. Saf. Sci..

[27] Naqvi H.M., Tiwari G. (2017). Factors contributing to motorcycle fatal crashes on National Highways in India. Int. J. Inj. Control Saf. Promot..

[28] Pai C.-W., Saleh W. (2008). Modelling motorcyclist injury severity by various crash types at T-junctions in the UK. Saf. Sci..

[29] Chung Y., Song T.-J., Yoon B.-J. (2014). Injury severity in delivery-motorcycle to vehicle crashes in the Seoul metropolitan area. Accid. Anal. Prev..

[30] Geedipally S.R., Turner P.A., Patil S. (2011). Analysis of Motorcycle Crashes in Texas with Multinomial Logit Model. Transp. Res. Rec. J. Transp. Res. Board.

[31] Wahab L., Jiang H. (2019). A multinomial logit analysis of factors associated with severity of motorcycle crashes in Ghana. Traffic Inj. Prev..

[32] Mannering F.L., Bhat C.R. (2014). Analytic methods in accident research: Methodological frontier and future directions. Anal. Methods Accid. Res..

[33] Chang F., Li M., Xu P., Zhou H., Haque M.M., Huang H. (2016). Injury Severity of Motorcycle Riders Involved in Traffic Crashes in Hunan, China: A Mixed Ordered Logit Approach. Int. J. Environ. Res. Public Health.

[34] Cunto F.J.C., Ferreira S. (2016). An analysis of the injury severity of motorcycle crashes in Brazil using mixed ordered response models. J. Transp. Saf. Secur..

[35] Lateef M.U. (2010). Estimation of Fatalities Due to Road Traffic Crashes in Karachi, Pakistan, Using Capture-Recapture Method. Asia Pac. J. Public Health.

[36] Behnood A., Mannering F.L. (2016). An empirical assessment of the effects of economic recessions on pedestrian-injury crashes using mixed and latent-class models. Anal. Methods Accid. Res..

[37] Cerwick D.M., Gkritza K., Shaheed M.S., Hans Z. (2014). A comparison of the mixed logit and latent class methods for crash severity analysis. Anal. Methods Accid. Res..

[38] Ahmadi A., Jahangiri A., Berardi V., Machiani S.G. (2020). Crash severity analysis of rear-end crashes in California using statistical and machine learning classification methods. J. Transp. Saf. Secur..

[39] Xiong Y., Mannering F.L. (2013). The heterogeneous effects of guardian supervision on adolescent driver-injury severities: A finite-mixture random-parameters approach. Transp. Res. Part B Methodol..

[40] Chang F., Xu P., Zhou H., Chan A.H.S., Huang H. (2019). Investigating injury severities of motorcycle riders: A two-step method integrating latent class cluster analysis and random parameters logit model. Accid. Anal. Prev..

[41] Behnood A., Mannering F. (2017). The effect of passengers on driver-injury severities in single-vehicle crashes: A random parameters heterogeneity-in-means approach. Anal. Methods Accid. Res..

[42] Washington S., Karlaftis M., Mannering F., Anastasopoulos P. (2020). Statistical and Econometric Methods for Transportation Data Analysis.

[43] Seraneeprakarn P., Huang S., Shankar V., Mannering F., Venkataraman N., Milton J. (2017). Occupant injury severities in hybrid-vehicle involved crashes: A random parameters approach with heterogeneity in means and variances. Anal. Methods Accid. Res..

[44] Train K. (2000). Halton Sequences for Mixed Logit.

[45] Pervez A., Huang H., Lee J., Han C., Li Y., Zhai X. (2022). Factors Affecting Injury Severity of Crashes in Freeway Tunnel Groups: A Random Parameter Approach. J. Transp. Eng. Part A Syst..

[46] Shaheed M.S.B., Gkritza K., Zhang W., Hans Z. (2013). A mixed logit analysis of two-vehicle crash severities involving a motorcycle. Accid. Anal. Prev..

[47] Greene W.H. (2012). Econometric Analysis.

[48] Washington S.P., Karlaftis M.G., Mannering F.L. (2010). Statistical and Econometric Methods for Transportation Data Analysis.

[49] Rezapour M., Mehrara Molan A., Ksaibati K. (2020). Analyzing injury severity of motorcycle at-fault crashes using machine learning techniques, decision tree and logistic regression models. Int. J. Transp. Sci. Technol..

[50] Lee J., Abdel-Aty M., Wang J.-H., Lee C. (2017). Long-Term Effect of Universal Helmet Law Changes on Motorcyclist Fatal Crashes: Comparison Group and Empirical Bayes Approaches. Transp. Res. Rec. J. Transp. Res. Board.

[51] Ali S., Khalid B., Kiani R.S., Babar R., Nasir S., Rehman N., Adnan M., Goheer M.A. (2019). Spatio-Temporal Variability of Summer Monsoon Onset over Pakistan. Asia-Pac. J. Atmos. Sci..

[52] Gumasing M.J.J., Magbitang R.V. Risk Assessment Model Affecting the Severity of Motorcycle Accidents in Metro Manila. Proceedings of the 2020 IEEE 7th International Conference on Industrial Engineering and Applications (ICIEA).

[53] Sari Y., Yudhistira M.H. (2021). Bad light, bad road, or bad luck? The associations of road lighting and road surface quality on road crash severities in Indonesia. Case Stud. Transp. Policy.

[54] Theofilatos A., Yannis G. (2015). A review of powered-two-wheeler behaviour and safety. Int. J. Inj. Control Saf. Promot..

[55] Jung S., Xiao Q., Yoon Y. (2013). Evaluation of motorcycle safety strategies using the severity of injuries. Accid. Anal. Prev..

[56] Aidoo E.N., Amoh-Gyimah R. (2019). Modelling the risk factors for injury severity in motorcycle users in Ghana. J. Public Health.

[57] Vajari M.A., Aghabayk K., Sadeghian M., Shiwakoti N. (2020). A multinomial logit model of motorcycle crash severity at Australian intersections. J. Saf. Res..

[58] Manan M.M.A., Várhelyi A., Çelik A.K., Hashim H.H. (2018). Road characteristics and environment factors associated with motorcycle fatal crashes in Malaysia. IATSS Res..

[59] Castro M., Paleti R., Bhat C.R. (2013). A spatial generalized ordered response model to examine highway crash injury severity. Accid. Anal. Prev..

[60] Xie Y., Zhao K., Huynh N. (2012). Analysis of driver injury severity in rural single-vehicle crashes. Accid. Anal. Prev..

[61] Jou R.-C., Yeh T.-H., Chen R.-S. (2012). Risk Factors in Motorcyclist Fatalities in Taiwan. Traffic Inj. Prev..

[62] Moskal A., Martin J.-L., Laumon B. (2012). Risk factors for injury accidents among moped and motorcycle riders. Accid. Anal. Prev..

[63] Khan U., Zia N., Awan S., Khan A. (2011). Perception of Pakistani women pillion riders about helmet use: A qualitative study. J. Epidemiol. Community Health.

[64] Siddiqui A.A., Shamim M.S., Jooma R., Enam S.A. (2006). Long scarf injuries. J. Coll. Physicians Surg. Pak..

[65] Schneider W.H., Savolainen P.T. (2011). Comparison of Severity of Motorcyclist Injury by Crash Types. Transp. Res. Rec. J. Transp. Res. Board.

[66] Syed W.H., Yasar A., Janssens D., Wets G. (2014). Analyzing the Real Time Factors: Which Causing the Traffic Congestions and Proposing the Solution for Pakistani City. Procedia Comput. Sci..

[67] Haque M.M., Chin H.C., Debnath A.K. (2012). An investigation on multi-vehicle motorcycle crashes using log-linear models. Saf. Sci..

[68] Pawar D.S., Patil G.R. (2017). Minor-Street Vehicle Dilemma While Maneuvering at Unsignalized Intersections. J. Transp. Eng. Part A Syst..

[69] Se C., Champahom T., Jomnonkwao S., Chaimuang P., Ratanavaraha V. (2021). Empirical comparison of the effects of urban and rural crashes on motorcyclist injury severities: A correlated random parameters ordered probit approach with heterogeneity in means. Accid. Anal. Prev..

[70] Budd L., Allen T., Newstead S. (2018). Current Trends in Motorcycle-Related Crash and Injury Risk in Australia by Motorcycle Type and Attributes.

